# A mixed-methods investigation of infant and young child feeding practices in rural Ethiopia: integrating insights from surveys, direct observation, and qualitative research

**DOI:** 10.3389/fnut.2026.1794352

**Published:** 2026-04-21

**Authors:** Ibsa Abdusemed Ahmed, Amanda Ojeda, Crystal Slanzi, Chhavi Tiwari, Jemal Yousuf Hassen, Arie H. Havelaar, Sarah McKune

**Affiliations:** 1 University of Florida, Gainesville, FL, United States; 2 Haramaya University, Dire Dawa, Ethiopia; 3 California State University, Los Angeles, CA, United States

**Keywords:** complementary feeding, direct observation, Ethiopia, exclusive breastfeeding, infant and young child feeding, prelacteal feeding, 24-hour recall

## Abstract

**Background:**

Optimal infant and young child feeding (IYCF) practices, particularly exclusive breastfeeding (EBF) for the first 6 months, are critical for child survival and development. In low- and middle-income countries, EBF prevalence is commonly estimated using maternal 24-h recall, which may overestimate true practices. This study quantified discrepancies between self-reported and directly observed feeding behaviors and explored socio-cultural determinants of IYCF in rural eastern Ethiopia.

**Methods:**

A sequential explanatory mixed-methods study was conducted among 106 mother-infant dyads with monthly maternal-recall surveys and two in-home observation sessions in a 79-infant subsample. Discrepancies were examined using chi-square or Fisher’s exact tests. To contextualize findings, 26 semi-structured interviews and five focus group discussions with caregivers and community influencers were conducted and thematically analyzed.

**Results:**

Cumulative 24 h recall across the first 6 months classified 70% of infants <6 months as EBF. Stricter WHO criteria reduced prevalence (18%; *p* = 0.002). Direct observation confirmed early supplementation: 77% of infants <6 months received non-breastmilk substances, 5% met WHO criteria under survey and observation. Surveys indicated timely complementary feeding (6–8 months) in 55% of infants, 30% introduced early, 15% late. Key EBF barriers included prelacteal feeding, perceptions of milk insufficiency, seasonal food insecurity, and elder influence. Traditional postpartum rest (*ulma*) facilitated early breastfeeding, but its protective role diminished as mothers resumed livelihoods.

**Conclusion:**

True EBF is markedly lower than 24 h recall estimates, highlighting the need for improved approaches and culturally tailored interventions that address feeding rituals, food insecurity, and elder’s social influence, while leveraging protective traditions like *ulma*.

## 1 Introduction

Optimal infant and young child feeding (IYCF) practices are fundamental to child survival, growth, and development (1, 2). The World Health Organization (WHO) recommends initiating breastfeeding within 1 h of birth, exclusive breastfeeding (EBF) for the first 6 months, and timely introduction of complementary foods at 6–8 months, with continued breastfeeding up to 2 years and beyond (3). Suboptimal IYCF practices contribute significantly to child morbidity, mortality, and malnutrition, accounting for over 800,000 preventable child deaths annually (2, 4).

Breastfeeding offers well-documented benefits: protection against diarrhea, pneumonia, asthma, obesity, and sudden infant death syndrome (5–9), improved cognitive development, and healthier eating habits. Mothers also benefit through reduced risks of breast and ovarian cancers, type 2 diabetes, and hypertension (4, 10–12).

Globally, approximately 48% of infants under 6 months are exclusively breastfed, with substantial regional disparities; one-third are introduced to complementary foods too early and one-fifth too late (3, 13, 14). WHO and UNICEF have set an ambitious global target of 70% EBF prevalence by 2030, yet most low- and middle-income countries remain off track (3).

Monitoring IYCF commonly relies on maternal 24-h recall due to its simplicity and low cost (15). However, this method is prone to recall and social desirability bias and may miss infrequent feeding events. It also cannot capture early-life practices such as prelacteal feeding, which disqualify infants from true EBF status under WHO’s definition (16). Consequently, EBF prevalence is often overestimated, especially in cultures where prelacteal feeding is common (17, 18). Direct observation can document actual feeding events more accurately and mitigate recall and social desirability bias (19, 20), but implementation is unfeasible in large study populations and is rarely integrated with surveys in low-resource settings.

Ethiopia has adopted several initiatives to improve IYCF practices, including the National Nutrition Program, the Baby-Friendly Hospital Initiative, and community-based breastfeeding support (21–26). HSTP II (27) set targets to raise EBF prevalence from 58 to 70% by 2025, ensure 80% of newborns are breastfed within 1 h, and maintain 75% continued breastfeeding at 12–15 months (27).

EBF prevalence increased from 49 to 59% between 2005 and 2019, yet remains below national targets (21). Rates decline sharply with infant age from 73% in the first month to about 40% by 4–5 months. Timely complementary feeding at 6–8 months is achieved by 66–69% of infants, while continued breastfeeding rates remain high (87% first year; 72% second year) (21). Early initiation of breastfeeding has stagnated around 73% and prelacteal feeding has increased nationally from 8 to 12% since 2016 (21). In some regions, prevalence is much higher, for example, 36% in Afar and 46% in Oromia (22, 23). These regional variations are linked to factors like antenatal counseling, place of delivery, and urban versus rural residence (28–30).

Multiple factors influence IYCF practices in Ethiopia, including cultural beliefs, maternal characteristics, healthcare access, and social norms (24–26, 28, 31–34). Traditional advice from elders often carries more weight than clinical recommendations in infant feeding decisions (29). The following sections focus on three key IYCF practices, colostrum avoidance, prelacteal feeding, and complementary feeding timing, each of which warrants specific attention in Ethiopian contexts.

Many Ethiopian communities have high colostrum feeding rates (>90% in parts of Amhara and Hararghe) (30, 35), however in other areas, a traditional practice of withholding colostrum poses health risks by depriving newborns of crucial nutrients and antibodies (30, 36). A recent meta-analysis estimated colostrum avoidance at 19% nationally, with mothers who lacked breastfeeding counseling being four times more likely to avoid colostrum (37) due to beliefs that colostrum is dirty, non-nutritious, or harmful (35, 36, 38). Thus, targeted counseling remains essential where avoidance persists (39, 40).

Prelacteal feeding rates remain high in several regions: 46.4% in Kersa District, 43.9% in Haramaya District, 29% in Oromia, and 27.4% in Amhara (11, 22, 40). This practice involves the provision of substances such as water (often with sugar), butter, or milk before breastfeeding is established, and is deeply embedded in cultural rituals across many Ethiopian communities (28–30). Such feeds are commonly perceived as cleansing or as blessings rather than nutrition, and are associated with male infants (reflecting cultural preferences), food insecurity, maternal education, and other socio-demographic factors (40). Prelacteal feeding increases the risk of neonatal infections, gastrointestinal issues, and neonatal mortality while interfering with early breastfeeding. Importantly, it is a primary driver of EBF failure in the first days of life (41).

Early (before 6 months) and late (after 8 months) introduction of complementary foods is common in Ethiopia. About 36% of infants receive complementary foods in the recommended 6–8 month window (42). Early introduction is often linked to perceived breast milk insufficiency, maternal workload, or misinformation (26, 31, 32). Late introduction may result from the belief that breast milk alone is sufficient or that infants are not yet ready for solid foods (43, 44).

Both patterns carry risks: early introduction increases infection and diarrhea risk, while late introduction can result in nutritional deficiencies and growth faltering (43, 45). Many complementary foods are cereal- or porridge-based with few animal-source foods or fruits/vegetables, largely due to cost constraints (46, 47). In some areas, unpasteurized animal milk is given to infants for perceived health benefits, despite risks of illness from raw milk (48, 49).

Haramaya District, the focus of this study, exemplifies persistent IYCF challenges. A 2018 formative survey reported that 52% of infants under 6 months were exclusively breastfed, yet 44% received prelacteal feeds and 14% experienced untimely complementary feeding (50). Prelacteal feeding emerged as the strongest predictor of suboptimal breastfeeding, alongside household food insecurity, khat cultivation, maternal literacy, and antenatal care access (50).

Given the persistence of prelacteal feeding and untimely complementary feeding in this region, and the negative impact on EBF rates, this study sought to provide a multi-faceted understanding of IYCF practices and their determinants in this community. By integrating quantitative and qualitative methods, we quantified discrepancies between reported and observed feeding behaviors and explored the cultural context shaping these practices.

## 2 Methods

### 2.1 Study design and setting

**Figure 1:**
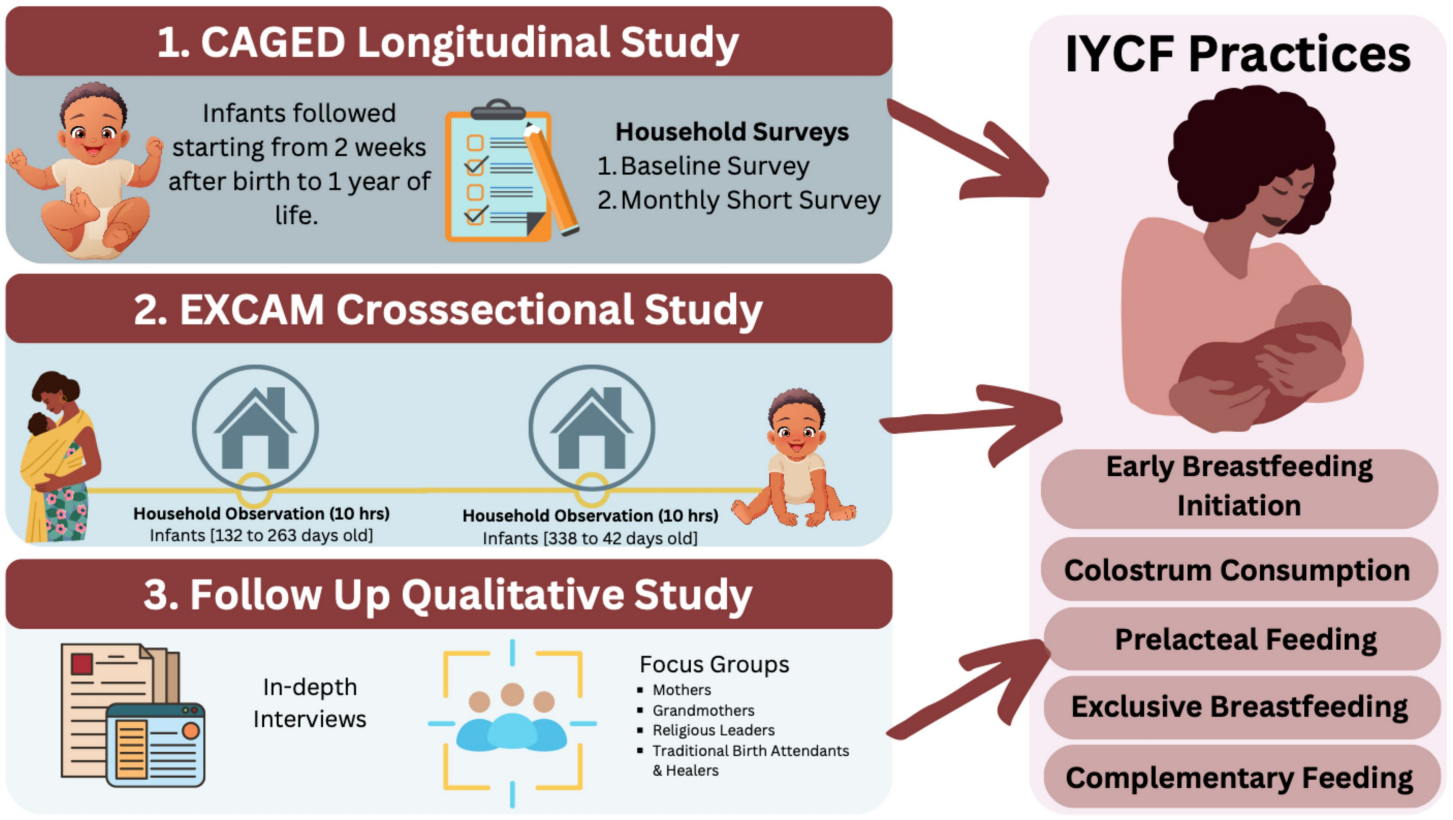
Analytical framework to assess infant young child feeding practices (IYCF) in Haramaya, Ethiopia. IYCF were evaluated by integrating maternal recall and direct observation data from two linked studies, CAGED and EXCAM, respectively, with interviews and focus group discussions with mothers and members of their community. Created using Canva.com.

We employed a sequential explanatory mixed-methods design in Haramaya District, Eastern Oromia, Ethiopia. Quantitative data were collected through structured household surveys and direct observation (December 2020–June 2022), followed by qualitative inquiry (January–February 2024) to contextualize the findings (Figure 1). The qualitative component was conducted after preliminary analysis of the quantitative and observational phases as a follow-up explanatory inquiry within the sequential mixed-methods design. It was intended to clarify the social and cultural factors underlying prelacteal feeding and other infant feeding behaviors observed in the main study. This inquiry was also informed by findings from the parent cohort, where prelacteal feeding was associated with increased Campylobacter load in the first month of life (51).

The quantitative phase drew on two linked studies: the Campylobacter Genomics and Environmental Enteric Dysfunction (CAGED) longitudinal birth cohort study (*n* = 106 mother–infant dyads) and the EXposure Assessment of CAMpylobacter Infection in Rural Ethiopia (EXCAM) nested observational study at two-time points within the CAGED study. Both projects ran from December 2020 to June 2022. CAGED collected baseline and monthly household survey data on infant feeding patterns, while EXCAM conducted two 10-h direct observation sessions on a subsample of 79 infants during individual home visits at 4–8 months and 11–14 months of age. Seventy-six infants completed both EXCAM observation rounds. CAGED details are published elsewhere (19, 52).

Haramaya District is an agrarian highland zone characterized by bimodal rainfall and predominantly rain-fed agriculture. The estimated population of 330,000 (53) depends mainly on sorghum and maize cultivation, with khat as the primary cash crop. Most residents speak Afaan Oromo, follow Islamic traditions, and face limited access to improved water sources (58%). Health services include one primary hospital, five health centers, and 38 health posts staffed by Health Extension Workers who are tasked with IYCF counseling (54).

### 2.2 Data collection

#### 2.2.1 Quantitative phase

- *Cohort Surveys*: Monthly structured interviews with 106 mother–infant dyads captured breastfeeding initiation, colostrum and prelacteal feeding, exclusive breastfeeding status (via 24 h recall), timing of complementary food introduction, feeding frequency, breastfeeding session duration, and consumption of non-breast milk liquids.
- *Direct Observations*: Trained field staff conducted two 10-h home observations for each infant using the Countee mobile application (v2.2.1) (55). Real-time data captured breastfeeding session duration, breastfeeding frequency, and presentation of non-breast milk substances. To minimize reactivity, observers first familiarized themselves with households and maintained a non-interventional stance during visits (19). Direct observations were conducted by trained Afaan Oromo-speaking field research assistants recruited from the local area, all of whom were trained in non-interventional household observation procedures before data collection; additional details on observer experience and the direct observation methods are provided elsewhere (19).

#### 2.2.2 Qualitative phase

We conducted 25 in-depth individual interviews with mothers, five with fathers, nine with grandmothers, and 12 with community leaders (including religious leaders, traditional healers, and health workers). Five focus group discussions (FGDs) were also held: three with mothers, one with fathers, and one with elder caregivers, each involving five participants and lasting 60–90 min. Participant characteristics are summarized in Table 1.

**Table 1:** Participant demographics in semi-structured interviews and FGDs.

Category	Participants (*n*)	Interviews (*n*)	FGDs (*n* groups; *n* participants)	Gender	Age (years)	Role in IYCF practices
Mothers	25	10	3 FGDs; 15 participants	Female	21–34	Primary caregivers for infant feeding
Fathers	5	0	1 FGD; 5 participants	Male	28–60	Support and influence feeding
Grandmothers	9	4	1 FGD; 5 participants	Female	47–55	Influence feeding through traditional wisdom
Traditional birth attendants	3	3	0	Female	30–55	Guide mothers during childbirth and postpartum
Religious leaders	3	3	0	Male	28–60	Reinforce cultural and spiritual IYCF practices
Traditional healers	3	3	0	Male	28–60	Influence traditional caregiving beliefs
Community health workers	3	3	0	Female	30–45	Educate & promote IYCF recommendations

All qualitative sessions were conducted in Afaan Oromo by trained bilingual facilitators, audio-recorded with consent, transcribed verbatim, and translated into English. Interview and FGD guides, informed by preliminary survey and observation findings, explored perceptions of colostrum, prelacteal feeding, breast milk adequacy, food introduction norms, exposure to health messages, and seasonal constraints on breastfeeding. The semi-structured interview guides and focus group discussion guides are provided in Supplementary material S1.

### 2.3 Measurements and definitions

#### 2.3.1 Self-reported indicators

We applied WHO definitions for infant feeding indicators (56), based on self-reported data:

- *EBF*: Infant receives only breast milk (except prescribed medicines or vitamins) during first 6 months.

- *Recall-based EBF*: mother reported no substances other than breastmilk in the past 24 h.
- *Composite EBF*: required no prelacteal feeds, initiation within first hour of birth, and no substances before 6 months across all monthly surveys.

- *Prelacteal feeding*: Any food or fluid given to a newborn before breastfeeding is established (typically within the first 3 days).
- *Timely initiation of breastfeeding*: Initiating breastfeeding within 1 h of birth.
- *Timely complementary feeding*: Introducing solid/semi-solid foods between 6 and 8 months.
- *Early introduction*: Complementary foods/liquids introduced before 6 months.
- *Late introduction*: Solid foods introduced after 8 months.
- *Breastfeeding frequency, duration, and inter-feed intervals (reported)*: Mothers were asked to estimate the average number of breastfeeding sessions per 24 h and the typical duration of a session.

#### 2.3.2 Direct observation indicators

Direct in-home observations provided real-time behavioral data on breastfeeding and supplementation practices, recorded using the Countee mobile application for standardized timestamping (55). Indicators included:

- *Breastfeeding frequency, duration, and inter-feed intervals*: Logged in real time with Countee for two 10-h visits per infant. Sessions lasting <10 min were defined as short based on evidence that very brief feeds may compromise milk transfer, particularly hindmilk rich in fat and energy (57). Although WHO does not prescribe a fixed duration, it highlights the need for both frequent feeds (8–12 per 24 h) and sufficient time to ensure effective foremilk–hindmilk transfer (6).
- *Observation window*. “Observed non-EBF” = any non-breastmilk intake during a single 10-h daytime visit. If none occurs, the infant is “observed EBF” within that window only (not a 24-h classification). Cross-validation with survey data was possible for the 39 infants under 6 months who contributed to both methods.

### 2.4 Data analysis

Quantitative data were analyzed using R software v4.3.1. We calculated descriptive statistics (proportions and confidence intervals) for key indicators and assessed differences between maternal reports and observational data using chi-square or Fisher’s exact tests (significance: *p* < 0.05). Age-stratified analyses (0–5 months vs. 6–11 months) examined changes in feeding frequency and supplementation.

Qualitative data underwent thematic analysis using Dedoose software (58). Two analysts independently developed a codebook reflecting key concepts from transcripts, including beliefs about colostrum, rationales for prelacteal feeding, perceptions of breast milk adequacy, family/cultural pressures, and responses to health messaging. Coding was refined through iterative comparison until thematic saturation was reached. Credibility was enhanced through investigator triangulation and member checking.

### 2.5 Integration of findings

Quantitative and qualitative results were synthesized during the interpretation phase. We identified points of convergence (where cultural explanations aligned with observed practices) and divergence (discrepancies between stated beliefs and behaviors) to develop a comprehensive understanding of IYCF practices in the study communities.

### 2.6 Ethics statement

The studies involving human participants were approved by the University of Florida Institutional Review Board (IRB201903141), the Haramaya University Institutional Health Research Ethics Committee (COHMS/1010/3796/20), and the Ethiopia National Research Ethics Review Committee (SM/14.1/1059/20). Written informed consent was obtained from all participating households (husband and wife), with mothers consenting to infant participation, using forms in the local language (Afan Oromo). The studies adhered to local legislation and institutional requirements.

## 3 Results

### 3.1 Quantitative results

#### 3.1.1 Self-reported IYCF practices

Survey data indicated nearly all mothers (95%; 93/98; 95% CI: 88.6–97.8) reported giving colostrum to their newborns. Sixty-eight percent (64/97; 95% CI: 56.1–74.6) reported early initiation of breastfeeding within 1 h and 67% (66/98; 95% CI: 57.6–75.8) practiced prelacteal feeding of something other than breast milk during the first 3 days of life.

**Figure 2:**
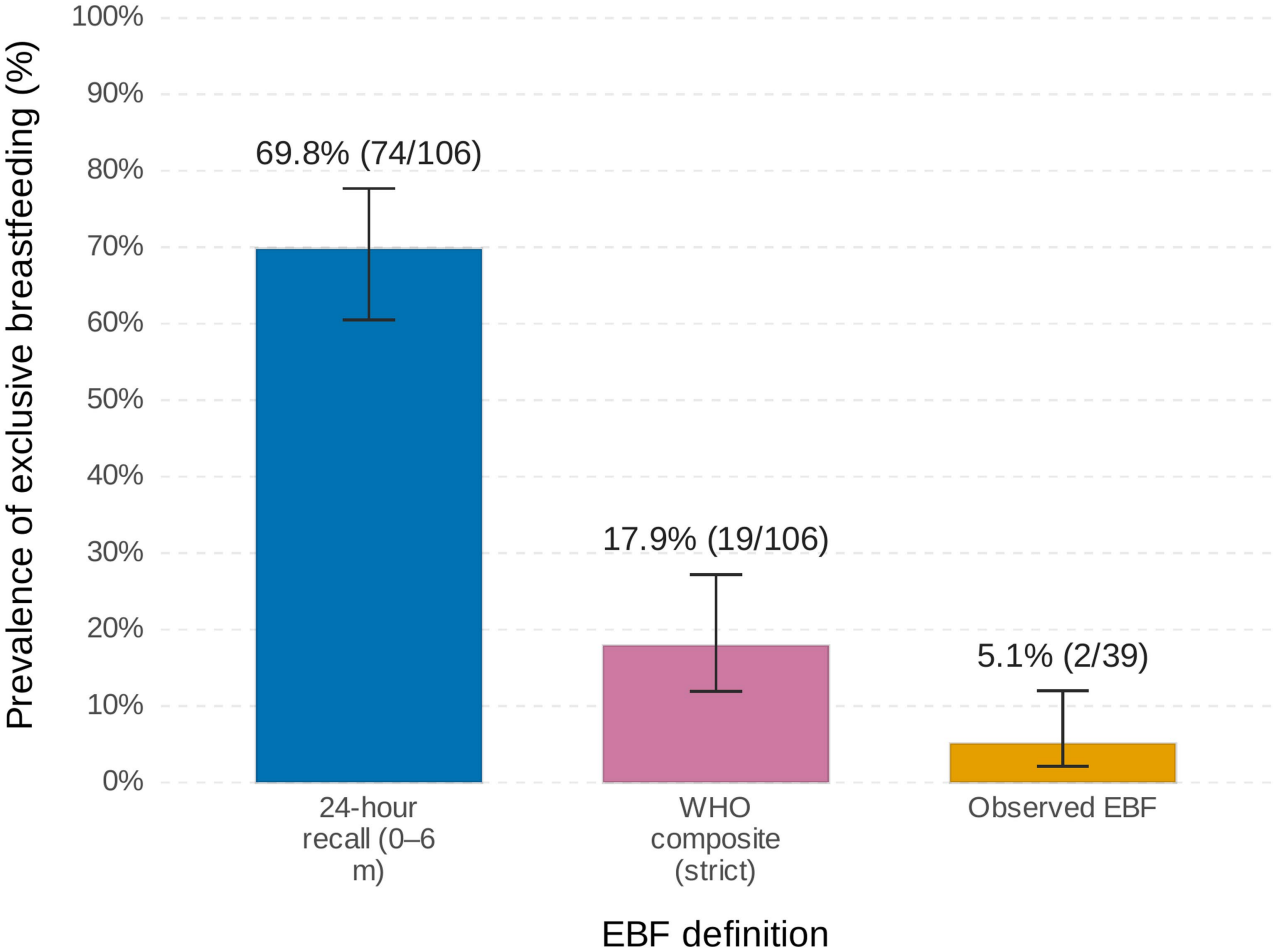
Exclusive breastfeeding (EBF) prevalence among infants aged 0–6 months in Haramaya, Ethiopia, by measurement method. Estimates with 95% confidence intervals.

The 24 h recall method revealed 69.8% of infants (74/106; 95% CI: 60.5–77.7) were classified EBF during their first 6 months. Applying the stricter WHO definition excluding infants receiving any prelacteal feeds, delayed initiation, or early introduction of complementary foods—reduced EBF prevalence to 18% (18/98; 95% CI: 11.9–27.2), a statistically significant decline (*χ*^2^ test, *p* = 0.002) (Figure 2).

**Figure 3:**
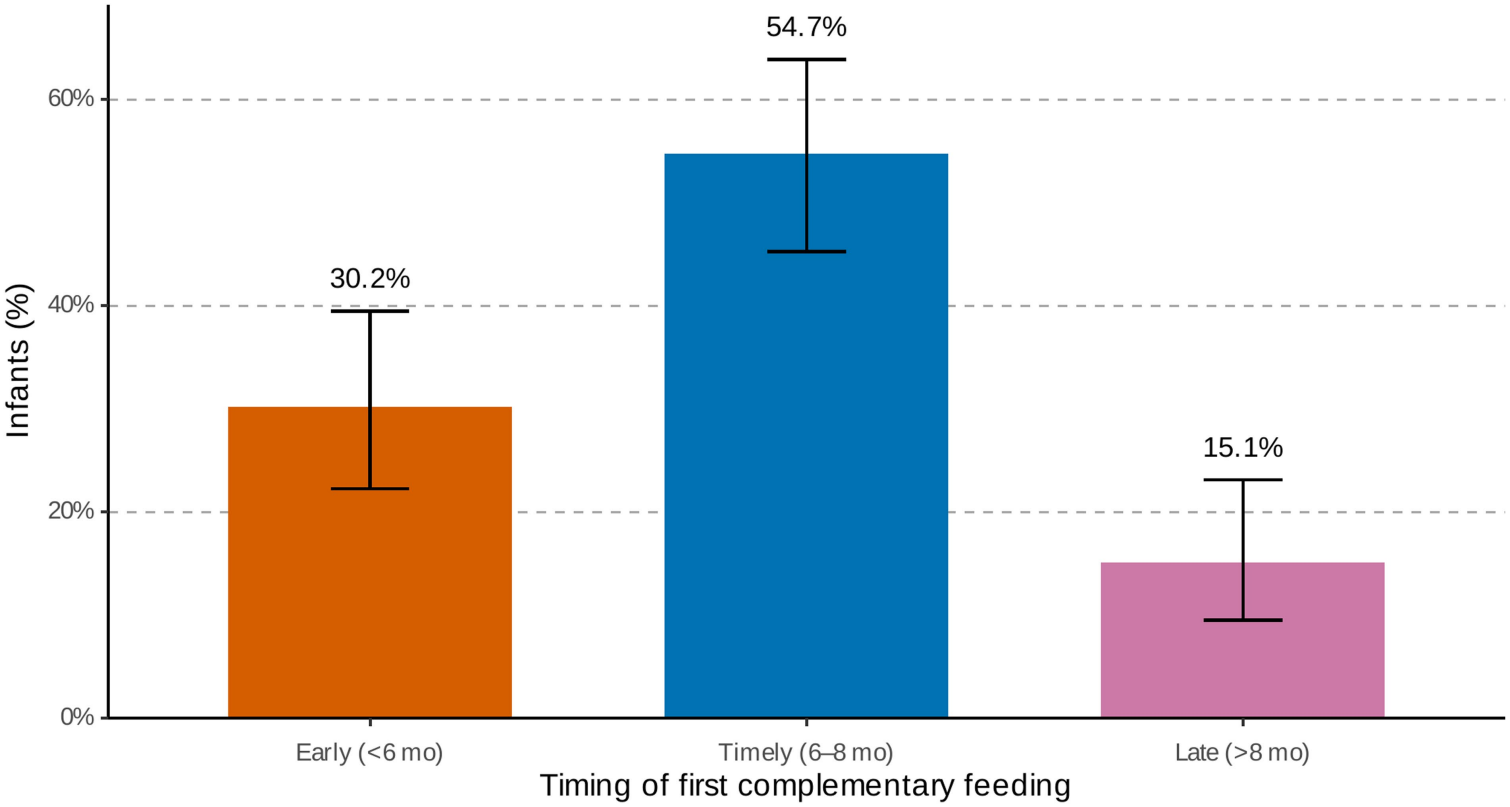
Timing of first complementary feeding among infants. Bars show point estimates with Wilson 95% confidence intervals for early (<6 months), timely (6–8 months), and late (>8 months) feeding categories.

Complementary feeding practices were suboptimal. Just over half of infants (54.7%; 58/106; 95% CI: 45.2–63.9) were introduced to solid or semi-solid foods at the recommended age of 6–8 months, while 45.3% (48/106; 95% CI: 36.1–54.8) experienced untimely introduction. Early feeding before 6 months was more common (67%; 32/48; 95% CI: 52.5–78.3) than late feeding after 8 months (33%; 16/48; 95% CI: 21.7–47.5). Most early introductions occurred between three and 6 months (Figure 3).

**Figure 4:**
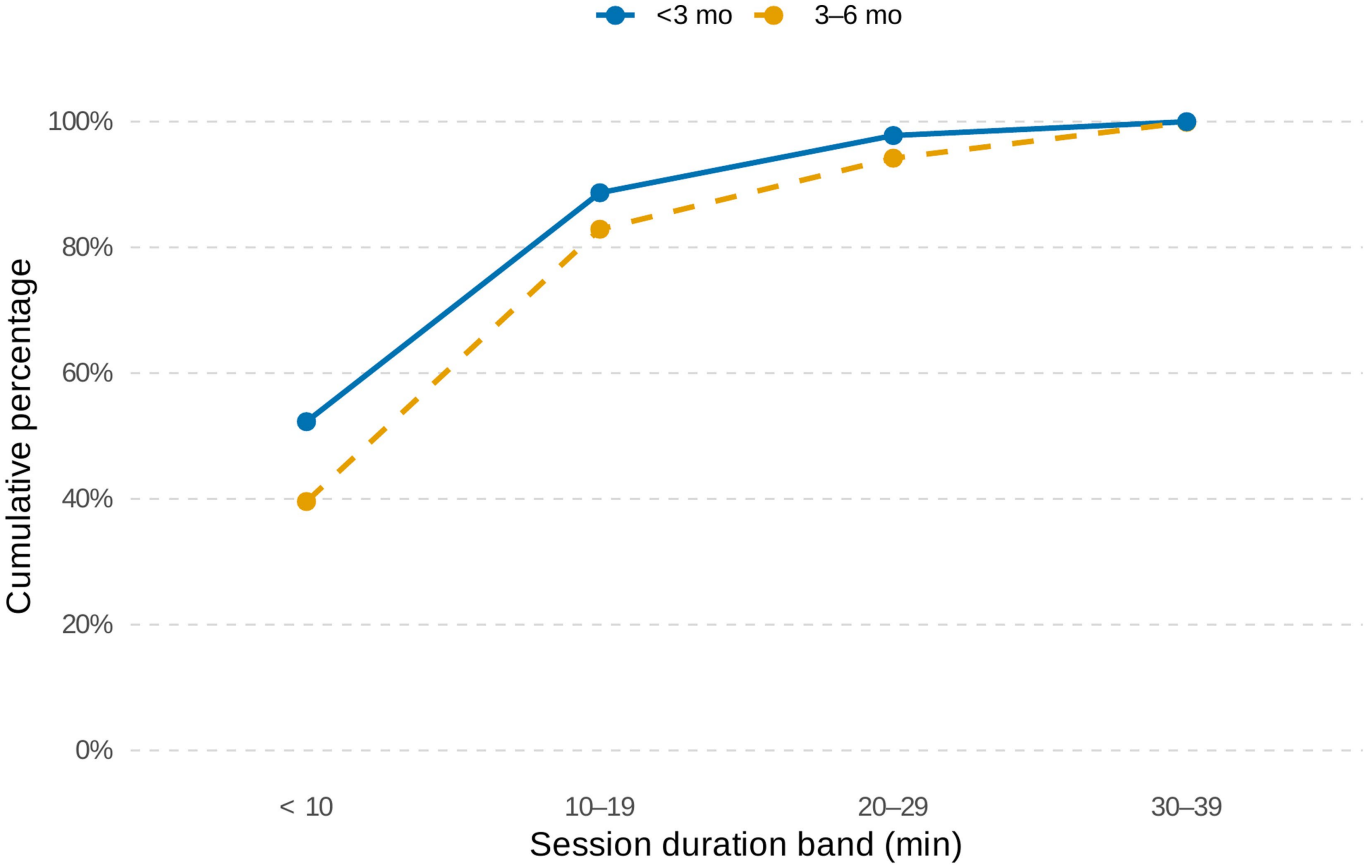
Cumulative distribution of breastfeeding session duration (24-h recall). Curves denote ages <3 month (solid blue) and 3–6 months (dashed gold) across bands in minutes.

Breastfeeding frequency and duration varied by infant age (Supplementary Table S1). Infants younger than 3 months were breastfed frequently in short sessions, often <10 min, with inter-feed intervals as short as 30 min. By three to 5 months, sessions became longer (10–19 min) and feeding frequency declined to 10–15 feeds per 24 h, compared with ≥15 feeds in younger infants (Figure 4).

#### 3.1.2 Antenatal care: breastfeeding counseling

Baseline survey data indicated that 79% of mothers (*n* = 77/97; 95% CI: 0.71–0.87) reported at least one ANC visit. Among those who received ANC, 54.5% (*n* = 42/77; 95% CI: 0.43–0.66) attended health posts, while 31.2% (*n* = 24/77; 95% CI: 0.21–0.42) attended health clinics.

Breastfeeding counseling during ANC was infrequent. Fewer than 5% of mothers reported receiving information on optimal breastfeeding practices. Approximately 95% (*n* = 73/77; 95% CI: 0.002–0.10) reported not being instructed on recognizing infant hunger cues, not being encouraged to feed on demand, and not being shown how to breastfeed or maintain lactation (Table 2).

**Table 2:** Reported breastfeeding and dietary counseling during antenatal care (baseline CAGED survey, *n* = 77).

Counseling topic	Response	*n* (%)
Breastfeeding practices:		
Taught to recognize infant hunger cues	Yes	4 (5.2)
	No	73 (94.8)
Encouraged to feed as often as infant desires	Yes	3 (3.9)
	No	74 (96.1)
Informed to breastfeed if breasts are full	Yes	4 (5.2)
	No	73 (94.8)
Encouraged to initiate breastfeeding within 1 h of birth	Yes	15 (19.5)
	No	62 (80.5)
Shown how to breastfeed and maintain lactation	Yes	4 (5.2)
	No	73 (94.8)

#### 3.1.3 Direct observation

**Figure 5:**
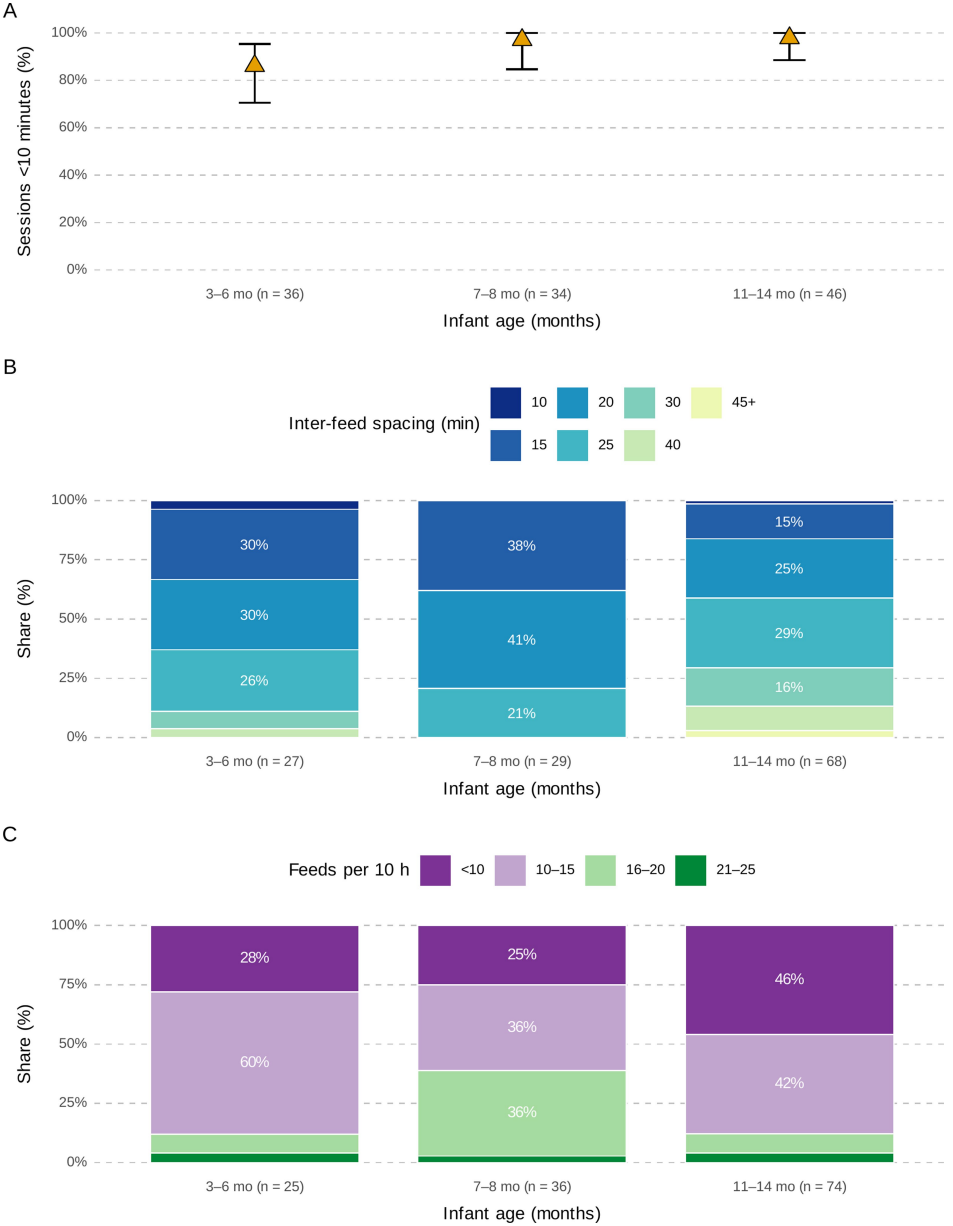
Breastfeeding session time characteristics observed during 10-h in-home visits in rural Eastern Ethiopia. **(A)** Proportion of breastfeeding sessions lasting <10 min by infant age group, with shorter sessions more common among older infants. **(B)** Distribution of inter-feed spacing (minutes), showing shorter intervals in younger infants and progressively longer intervals with age. **(C)** Number of breastfeeding sessions per 10-h observation, with younger infants feeding more frequently, declining steadily as age increased.

Direct observation confirmed overall feeding patterns while providing detailed behavioral insights (Supplementary Table S1). With increasing infant age, breastfeeding sessions became shorter, inter-feed intervals lengthened, and daily feeding frequency declined (Figure 5).

During the first observation (infants 3–8 months), more than half of breastfeeding episodes among 4–6-month-olds lasted <10 min. By 11–14 months, nearly all sessions were <10 min. Feeding frequency in older infants declined from 8 to 12 feeds per 24 h exhibited by 3–6-month-olds (Figure 5).

Complementary feeding was prevalent. Among 79 infants observed, 87% (69/79; 95% CI: 80.0–94.7) consumed at least one non-breastmilk item during the 10-h observation. The youngest infant observed receiving complementary food was 132 days (~4.3 months) old. Among infants under 6 months (*n* = 39), 77% (30/39; 95% CI: 63.7–90.1) were given non-breastmilk substances during the observations.

Among the 69 infants observed consuming complementary foods, water was most common (74%; 51/69; 95% CI: 63.6–84.2), followed by injera (48%; 33/69; 95% CI: 36.0–59.6). One infant consumed raw plant material, likely exploratory.

For the subgroup with both survey and observation data under 6 months (*n* = 39), maternal recall classified 54% (21/39; 95% CI: 38.2–69.4) as EBF, however, 67% (14/21; 95% CI: 46.5–86.8) of these reportedly EBF infants were observed receiving complementary foods. Ultimately, 18% (7/39; 95% CI: 5.9–29.9) were EBF by both recall and direct observation methods while just 5% (2/39; 95% CI: 0.0–12.0; Figure 2) met WHO criteria with no complementary feeding observed.

**Figure 6:**
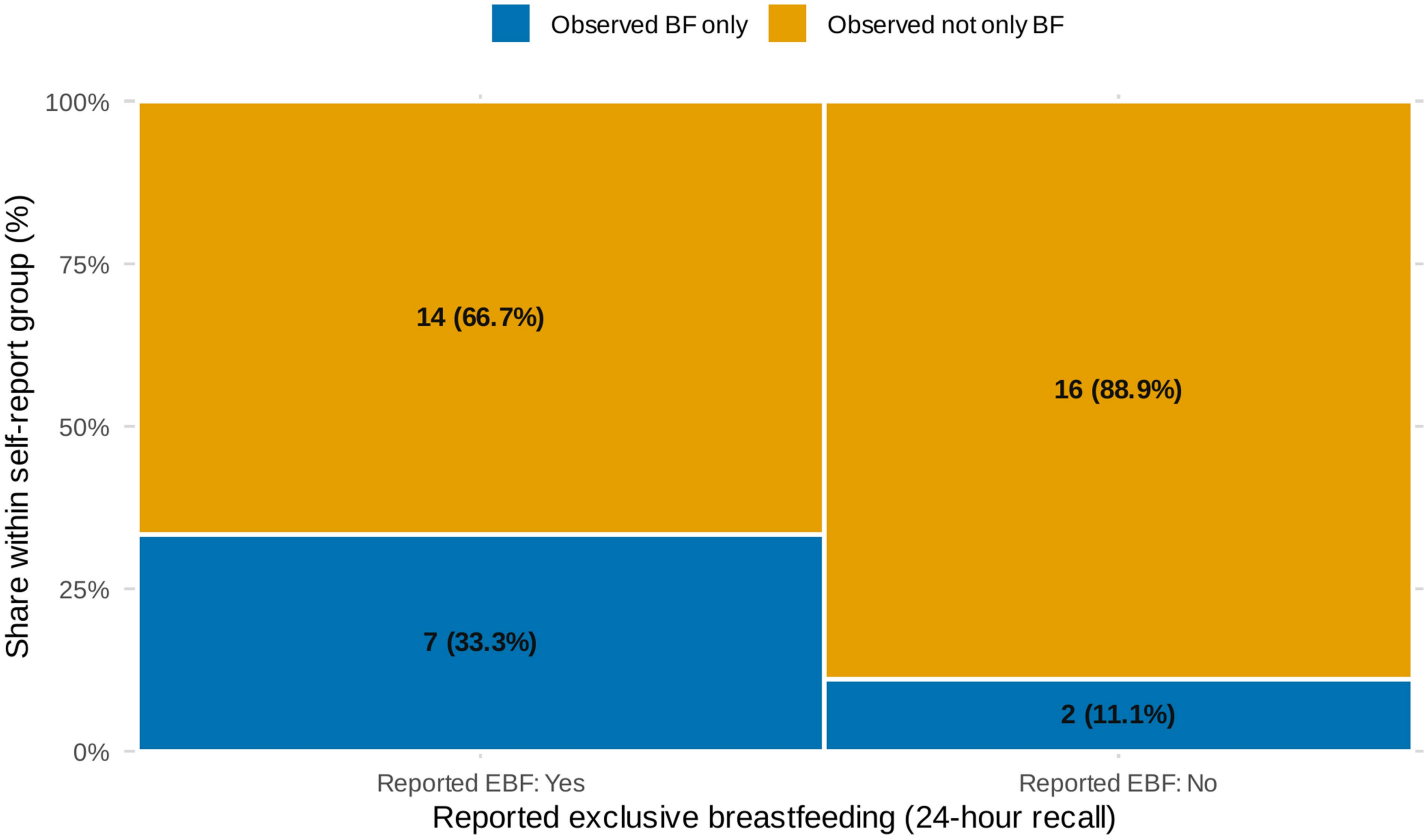
Reported versus observed exclusive breastfeeding (EBF) among infants aged 0–6 months (paired *n* = 39). Within each 24-h recall group, stacked bars show the proportion observed as EBF during a 10-h home observation (“Observed BF only,” blue) versus not exclusively breastfed (“Observed not only BF,” gold).

No significant differences were detected in observed complementary feeding between mothers who self-reported EBF and those who did not (*p* = 0.24 at first observation; *p* = 0.25 at second). By the second observation round (infants 11–14 months), complementary feeding was universal (100%; 76/76; 95% CI: 95.2–100). Within this group, 79% (60/76; 95% CI: 69.7–88.1) consumed water and 51% (39/76; 95% CI: 40.1–62.5) ate injera. All older infants continued breastfeeding at least occasionally (Figure 6).

#### 3.1.4 Attitudes and beliefs about IYCF

**Figure 7:**
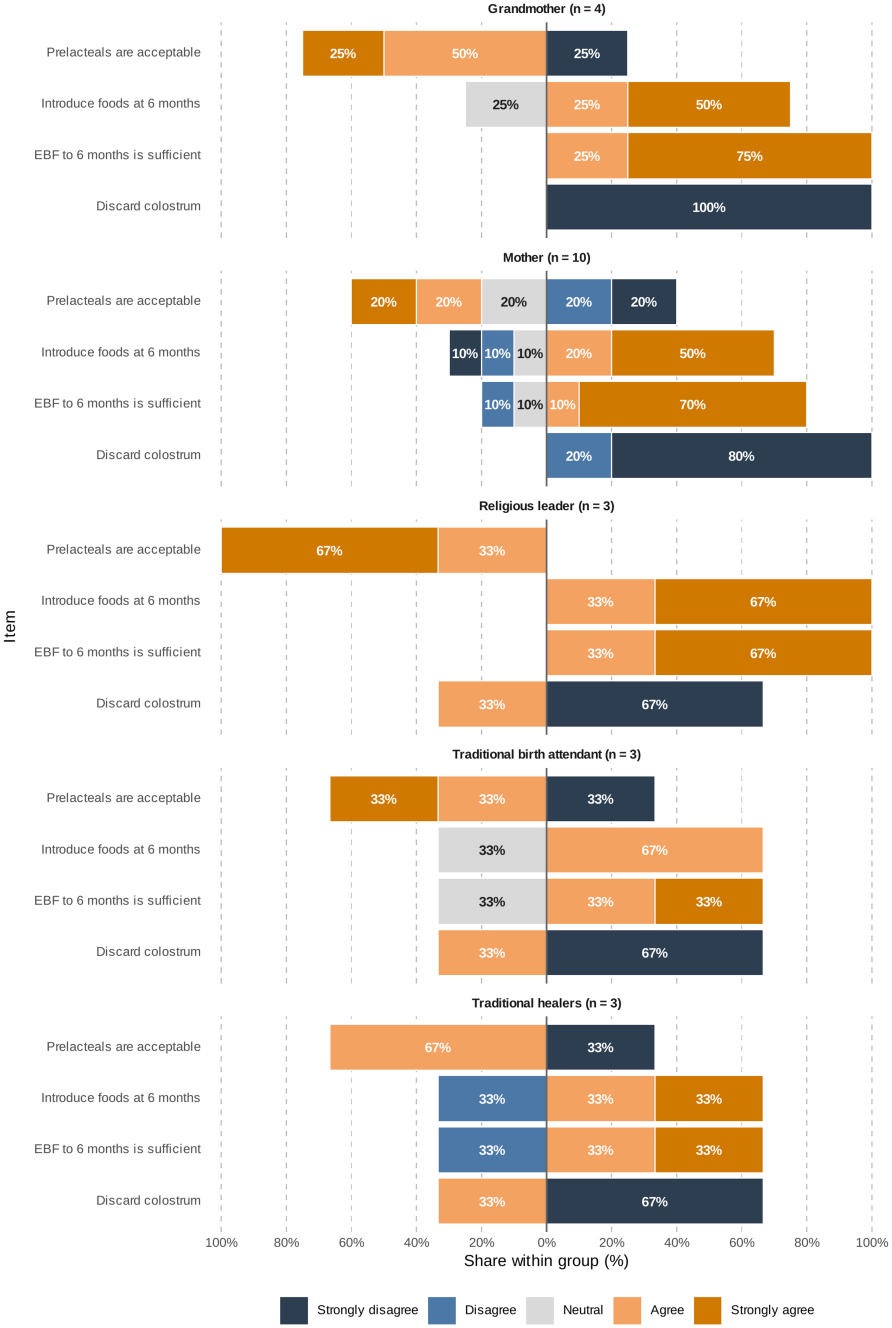
Attitudes toward IYCF by stakeholder group. Diverging stacked bars show within-group Likert responses to four items: prelacteals, colostrum, introducing foods at 6 months, and sufficiency of EBF to 6 months. Right of zero = IYCF-aligned (agree for timely introduction/EBF sufficiency; disagree for prelacteals/colostrum discard); left (including Neutral) = suboptimal. Percentages are shown on bars; totals may not equal 100% due to rounding. EBF, exclusive breastfeeding.

Caregivers’ attitudes toward IYCF were assessed through structured interviews and FGDs using Likert-scale items. The proportion of respondents in each stakeholder group (Figure 7) who expressed agreement or disagreement with key feeding statements highlight both strong areas of consensus and points of divergence shaped by cultural norms.

Colostrum feeding: Support for colostrum feeding was nearly universal. Most mothers (80%) and all grandmothers (100%) strongly disagreed with discarding colostrum. Community leaders also expressed unanimous support, with no participants advocating colostrum disposal.

Prelacteal feeding: Opinions were divided. Among mothers, 40% opposed the practice, 40% expressed some degree of support, and 20% were neutral. Endorsement was higher among traditional healers (67%) and religious leaders (50%), reflecting stronger acceptance of prelacteal feeding among these groups compared with mothers.

Breast milk sufficiency (0–6 months): Broad consensus emerged that breast milk alone is sufficient for the first 6 months. Mothers and grandmothers showed the strongest agreement (70–75% strongly agreed), while a small proportion of traditional birth attendants and healers expressed uncertainty.

Introduction of complementary foods (6 months): Support for timely introduction was strong. Across all participants, 43% strongly agreed with introducing complementary foods around 6 months, and most of the remainder moderately agreed. Endorsement was particularly high among traditional birth attendants and healers, with 67% strongly supporting timely introduction.

Perceptions of health education: Views were mixed. About 30% of participants agreed that health education improved their knowledge, while 26% traditional healers disagreed, suggesting perceived limited impact among some traditional actors.

### 3.2 Qualitative findings

The qualitative data from FGDs and SSIs provided deeper insights into the cultural logics, lived experiences, and barriers shaping IYCF. Eight interrelated themes emerged: (1) prelacteal feeding rituals, (2) postpartum resting practices *ulma*, (3) perceptions of EBF and early complementary feeding, (4) other culturally significant feeding customs, (51) economic constraints, (5) seasonal food insecurity, (6) sociocultural influences, and (7) knowledge gaps. We present a culturally grounded account of three critical practices—prelacteal feeding, *ulma*, and exclusive breastfeeding, followed by an integrated discussion of cross-cutting barriers. For details, see Supplementary Table S2.

#### 3.2.1 Prelacteal feeding practices

Prelacteal feeding was widely described as a deeply rooted cultural ritual. Families reported giving newborns water, sugar-water, or melted butter before breast milk is established. These substances were viewed as symbolic rather than nutritional. One mother explained:

*“In our tradition, offering water before breastmilk signifies the beginning of life. We say in Afaan Oromo, ‘areera male hin du’ani,’ which means, ‘a human being dies the day they are born.’ Giving water protects life from that moment.”* Many participants described these substances as protective or cleansing. One mother in an FGD shared: *“We give water or butter because it is believed to cleanse the baby’s stomach and prevent sickness.”*

Another noted the perceived strength-giving quality of butter: *“When we put butter on a baby’s tongue, it helps them grow strong and keeps their throat healthy.”* There were also gendered variations in ritual. A grandmother explained: *“Traditionally, if the newborn is a boy, we give him water on the first day. For a girl, milk mixed with sugar is thought to make her stronger.”*

Despite the persistence of these practices, shifts were visible among younger parents. One mother explained: *“I now avoid giving my newborn water because the health worker told me it is not good for the baby’s stomach.”* Similarly, a father noted: *“We have started telling our mothers and wives that prelacteal feeding is not necessary if breastmilk is available.”* Yet mothers consistently reported strong pressure from elders. One summarized: *“Even if I want to follow the advice of the health worker, my mother-in-law insists we give butter or water first. It is very hard to refuse.”*

#### 3.2.2 Postpartum resting tradition (*ulma*)

The postpartum resting period, locally known as *ulma*, was described as one of the most supportive practices for maternal and infant health. Mothers are relieved from chores for about 40 days, receiving warmth, care, and nutrient-rich foods like *shuro dhalinsaa* (fortified porridge) and butter. One mother described: *“During ulma, I was given porridge with butter and told to rest. My sisters and neighbors helped me with chores. It was the only time I could focus only on my baby.”* Fathers also acknowledged participating: *“During the ulma period, I make sure my wife gets enough rest. I help with housework so she can breastfeed and recover.”*

The practice is viewed as essential for restoring maternal strength and promoting milk flow. Warmth was emphasized as protective: *“We keep the mother warm, sometimes with a fire or blankets, because cold is believed to harm her and reduce milk.”* At the end of *ulma*, families celebrate *ulma baha*, a ritual marking the mother’s return to normal life. A grandmother described: *“We have a ceremony called ulma baha we clean the house, prepare food, and the mother dresses beautifully to show she is strong again.”*

Participants widely credited *ulma* with enabling proper breastfeeding during the early weeks. Yet once the resting period ends, mothers resume agricultural work and the intensive support fades. One mother reflected: *“After ulma, I had to go back to the farm. That is when it became difficult to breastfeed exclusively.”*

#### 3.2.3 Exclusive breastfeeding and early complementary feeding

Breastfeeding was described as universal and natural. One father said simply: *“In our culture, every mother breastfeeds. It is her duty.”* However, strict exclusive breastfeeding for 6 months was rarely achieved. The most common concern was perceived insufficiency of breast milk. One mother explained: *“By three months, the baby cries too much. I give him milk or porridge to make him full.”* Others linked EBF with maternal weakness, especially under conditions of food insecurity: *“If exclusive breastfeeding happens without a balanced diet, the mother becomes weak and malnourished. Sometimes we have to buy milk to give the baby.”*

Health workers confirmed these perceptions: *“Mothers tell us they fear exclusive breastfeeding will make them too weak to work or care for their other children, so they stop early.”* Elders, especially grandmothers were described as decisive in feeding choices. A young mother recounted: *“If my baby is crying and I have work to do, my mother-in-law will say the baby is hungry and I must give something. It is hard to refuse.”*

#### 3.2.4 Barriers to optimal IYCF practices

The narratives revealed five interrelated barriers shaping IYCF:

- Economic constraints: Poverty limited maternal diets and confidence in milk sufficiency. One father stated: *“Richer families do not worry about breastfeeding, but poor families struggle.”* Some mothers turned to formula, though costly and often diluted: *“Formula is expensive. If we cannot afford it, we give water or biscuits.”*
- Seasonal food insecurity: Agricultural cycles shaped breastfeeding capacity. *“During the dry season, food is scarce. Even our cows do not give milk. That is when we start giving other things to the baby.”*
- Sociocultural influence: Elders’ authority often outweighed professional advice. One mother shared: *“Feeding decisions are not mine alone. My mother-in-law decides when the baby should eat other food.”*
- Knowledge gaps: Few mothers received antenatal breastfeeding counseling. A participant reflected: *“I did not get any formal education on breastfeeding. I only learned from my family.”*
- Maternal workload: After *ulma*, women returned to strenuous labor. *“I leave my baby with relatives when I go to the farm. They often give him other food.”*

These barriers were mutually reinforcing: poverty and food insecurity undermined maternal nutrition and milk supply; workload constrained breastfeeding opportunities; and sociocultural authority limited adoption of health messages. Yet, examples of positive deviance emerged. Some mothers successfully practiced EBF, often with strong family support and better food security. These cases suggest interventions grounded in supportive traditions such as *ulma* and extended family engagement could improve outcomes.

## 4 Discussion

This mixed-methods study provides an integrated understanding of IYCF practices in rural eastern Ethiopia through longitudinal survey data with repeated direct observations, qualitative interviews, and FGDs. Triangulation of these complementary data sources enabled quantification, objective verification, and exploration of the cultural context shaping feeding behaviors and their determinants.

Our findings substantiate past evidence demonstrating the limitations of survey-based self-reported breastfeeding behavior and also quantifies these limitations in a specific population in Ethiopia. In our study, maternal 24-h recall substantially overstated exclusive breastfeeding when compared with stricter composite criteria and direct observation. This is an important methodological message for studies that rely on single cross-sectional surveys, particularly in low-resource settings where 24-h recall is commonly used to estimate EBF. Our findings support the need for updated approaches to measuring EBF and, where feasible, the integration of direct observations into public health research. The implications of understanding the true rate of EBF are critical in resource limited settings, where EBF not only ensures early protection from disease through maternal antibodies and optimal nutrition through breastmilk composition, but also protects infants from early exposure via foods and drinks to pathogens, which have been shown to colonize the guts of infants in this study population at a very high rate (59).

Scientific consensus is strong that EBF is best for optimal nutrition of children, yet rates remain low for myriad reasons. While early introduction of complementary foods and taboos around colostrum consumption have received extensive attention in maternal and child research, our research in Eastern Hararghe Zone, Ethiopia found prelacteal feeding responsible for 67% of mothers defaulting out of EBF, and the practice was supported by at least two-thirds of the community leaders, grandmothers, and traditional birth attendants we interviewed. This deeply engrained cultural practice, along with information about the importance of timely complementary feeding, should be targeted through behavior change communication programming during ANC visits, and post-partum and well-child exams. Public health efforts that target culturally embedded and religiously sanctioned activities must be approached with sensitivity and care and working with the community to ensure stakeholders understand the risk of the behavior and are part of developing a community-based response is likely to yield more lasting results.

A major contribution of this research is to understanding the detailed behavior of breastfeeding timing and duration among infants. Very little is known about the habits of breastfeeding within the home in LMICs as this behavior is hidden from public life. Using direct observations of women for 10 h in the first 6 months of their child’s life and another 10-h block in the second 6 months of the child’s life, allowed us to quantify and compare patterns of breastfeeding duration. These findings are important, as many women in these communities reported complementary feeding their children due to their perception of their own inadequate milk supply. Given the important compositional differences between foremilk and hindmilk, ensuring that women are breastfeeding completely draining each breast during a sitting can improve satiety among infants and may contribute to women’s perception of breastmilk sufficiency, which may in turn decrease untimely complementary feeding.

Our results also illuminate pathways linking poverty, food insecurity, and maternal workload to perceived milk insufficiency. The postpartum resting tradition, *ulma* functioned as a locally meaningful postpartum support period during which mothers described receiving rest, special foods, and practical help from husbands, sisters, relatives, and neighbors, which appeared to support breastfeeding in the early postpartum period. This finding is consistent with broader literature describing postpartum traditions that include maternal rest, prescribed foods, and organized household support (60). However, in our setting, this protection was temporary and diminished once women resumed agricultural labor, at which point competing work demands and separation from the infant often contributed to early supplementation. This pattern is consistent with evidence that return to work and inadequate structural support can undermine continued exclusive breastfeeding. In Ethiopia, Labour Proclamation No. 1156/2019 provides 120 days of paid maternity leave, but this remains shorter than the recommended 6 months of exclusive breastfeeding and offers limited protection for many women working in agricultural and informal settings (61, 62). Although neighbor support during postpartum rest is not widely documented in the Ethiopian IYCF literature, our finding is consistent with broader evidence that family and community support can facilitate breastfeeding continuation (29, 63). Leveraging and strengthening this existing support structures may therefore be more feasible and culturally appropriate than relying solely on longer-term socioeconomic change. In addition, the majority of mothers attended at least one ANC visit, yet our findings overwhelmingly suggest IYCF education falls short and opportunities to deliver targeted IYCF counseling are being missed. Strengthening the content and delivery of ANC could represent a critical leverage point for improving breastfeeding measures and empowering both mothers and key household influencers is essential to sustaining optimal feeding (29).

The geographic focus on rural eastern Ethiopia limits generalizability of this study, as does the observations at only two discrete time points, which may not capture seasonal or developmental variability in feeding. While observer reactivity likely diminished, its influence cannot be fully ruled out. Finally, the sample size was sufficient for detecting broad trends but not for rarer practices or nuanced subgroup analyses. Future studies should expand to other regions and incorporate multi-seasonal observations across different child age stages to enhance generalizability.

## 5 Conclusion

This mixed-methods study highlights critical discrepancies between reported and observed IYCF practices in rural Ethiopia, shaped by deep cultural traditions and socioeconomic constraints. Interventions that are contextually grounded and multi-sectoral must address prelacteal feeding rituals and engage elders, household decision-makers, and local leaders as active change agents. Addressing these challenges requires culturally sensitive, integrated interventions that target both behavioral and structural determinants to sustainably improve child nutrition outcomes.

## Data Availability

The original contributions presented in the study are included in the article/Supplementary material, further inquiries can be directed to the corresponding author.
