# Supplementary material for: A mixed-methods investigation of infant and young child feeding practices in rural Ethiopia: integrating insights from surveys, direct observation, and qualitative research
Source: Front Nutr. 2026 Apr 21;13:1794352. doi: 10.3389/fnut.2026.1794352 (PMC13138919; doi:10.3389/fnut.2026.1794352)
Supplement: Supplementary file 1 [file Table_1.docx]

Supplementary Table S1

Supplementary Table S1. Assessment of breastfeeding practices via CAGED surveys (self-report) and direct observations (EXCAM).

| Study | CAGED Survey | CAGED Survey | EXCAM Observations | EXCAM Observations | EXCAM Observations |
| --- | --- | --- | --- | --- | --- |
| Study | Baseline % (n=106) | Baseline % (n=106) | First % (n=79) | First % (n=79) | Second % (n=76) |
| Infant Age | <3 months | 3-6 months | 3-6 months | 7-8 months | 11-14 months |
| Duration per BF session (minutes) | (n=44) | (n=53) | (n=36) | (n=34) | (n=46) |
| <10 | 52.3%(23/44) | 39.6%(21/53) | 86.1%(31/36) | 97.1%(33/34) | 97.8%(45/46) |
| 10-19 | 36.4%(16/44) | 43.3%(23/53) | 11.1%(4/36) | 2.9%(1/34) | 2.2%(1/46) |
| 20-29 | 9.1%(4/44) | 11.3%(6/53) | 2.8%(1/36) | 0 | 0 |
| 30-39 | 2.3%(1/44) | 5.7%(3/53) | 0 | 0 | 0 |
| Spacing between BF sessions (minutes) | (n=28) | (n=24) | (n=27) | (n=29) | (n=68) |
| 10 | 10.7%(3/28) | 0 | 3.7%(1/27) | 0 | 1.5%(1/68) |
| 15 | 10.7%(3/28) | 12.5%(3/24) | 29.6%(8/27) | 37.9%(11/29) | 14.7%(10/68) |
| 20 | 14.3%(4/28) | 20.8%(5/24) | 29.6%(8/27) | 41.4%(12/29) | 25%(17/68) |
| 25 | 0 | 4.2%(1/24) | 25.9%(7/27) | 20.7%(6/29) | 29.4%(20/68) |
| 30 | 53.6%(15/28) | 41.7%(10/24) | 7.4%(2/27) | 0 | 16.2%(11/68) |
| 40 | 0 | 12.5%(3/24) | 3.7%(1/27) | 0 | 10.3%(7/68) |
| 45+ | 10.7%(3/28) | 8.3%(2/24) | 0 | 0 | 2.9%(2/68) |
| Frequency of BF within 24hr (sessions) | (n=45) | (n=53) | (n=25) | (n=36) | (n=74) |
| <10 | 24.4%(11/45) | 9.4%(5/53) | 28%(7/25) | 25%(9/36) | 45.9%(34/74) |
| 10-15 | 53.3%(24/45) | 60.4%(32/53) | 60%(15/25) | 36.1%(13/36) | 41.9%(31/74) |
| 16-20 | 17.8%(8/45) | 30.2%(16/53) | 8%(2/25) | 36.1%(13/36) | 8.1%(6/74) |
| 21-25 | 4.4%(2/45) | 0 | 4%(1/25) | 2.8%(1/36) | 4.1%(3/74) |
