# Supplementary material for: A mixed-methods investigation of infant and young child feeding practices in rural Ethiopia: integrating insights from surveys, direct observation, and qualitative research
Source: Front Nutr. 2026 Apr 21;13:1794352. doi: 10.3389/fnut.2026.1794352 (PMC13138919; doi:10.3389/fnut.2026.1794352)
Supplement: Supplementary file 2 [file Table_2.docx]

Supplementary Table S2

Supplementary Table S2. Taxonomy of emergent themes surrounding existing IYCF practices and barriers to optimal IYCF practices, as identified through focus group discussions and semi-structured interviews.

| Themes | Subthemes | Quotes |
| --- | --- | --- |
| Prelacteal Feeding | Cultural beliefs and feeding practices | "We give water mixed with sugar as a prelacteal to soothe the baby before milk production begins" (in depth Interview Mother)​ |
| Prelacteal Feeding | Cultural beliefs and feeding practices | "We believe that giving water to a newborn ensures a smooth transition to breastfeeding" (FGD 5, Fathers) |
| Prelacteal Feeding | Cultural beliefs and feeding practices | "Newborns are given butter on their tongues so that they grow strong, and their throats stay healthy" (FGD 3, Mothers). |
| Prelacteal Feeding | Cultural beliefs and feeding practices | "We give water or butter because it is believed to cleanse the baby's stomach and prevent sickness" (FGD 1, Mothers). |
| Prelacteal Feeding | Cultural beliefs and feeding practices | "In our tradition, offering water [before breastmilk] signifies the beginning of life, as reflected in our saying 'areera male hin du'ani'" (FGD 1, Mothers). |
| Prelacteal Feeding | Cultural beliefs and feeding practices | "It is part of our culture to give the baby something sweet on their tongue as a blessing" (FGD 4, Grandmothers). |
| Prelacteal Feeding | Cultural beliefs and feeding practices | "Offering water [before breastmilk] signifies the beginning of life, as reflected in our saying 'areera male hin du'ani'" (FGD 1, Mothers) |
| Prelacteal Feeding | Gendered feeding norms | "Traditionally, if a newborn was male, it was believed that giving him water and female milk on the first day was important" (In-depth Interview, Grandmother). |
| Prelacteal Feeding | Gendered feeding norms | "For girls, we sometimes give milk mixed with sugar, as this is believed to make them stronger" (FGD 4, Grandmothers). |
| Prelacteal Feeding | Changing norms | “I now avoid giving my newborn water because the health worker told me it isn’t good for the baby’s stomach" (FGD 3, Mothers). |
| Prelacteal Feeding | Changing norms | "We have started telling our mothers and wives that prelacteal feeding is not necessary if breastmilk is available" (FGD 5, Fathers). |
| Postpartum Resting Practices | Postpartum care rituals (‘ulma’) prioritize maternal recovery and bonding. | "During ulma, mothers are kept warm and given nourishing foods like porridge and butter to recover their strength" (FGD 1, Mothers). |
| Postpartum Resting Practices | Families provide financial and emotional support during the postpartum recovery period. | "We collect contributions to support mothers during their resting period" (FGDs 3–5, Mothers)​ |
| Postpartum Resting Practices | Fathers play supportive roles during the postpartum period. | "During the ulma period, I ensure my wife gets enough rest and help with household chores" (FGD 5, Fathers)​ |
|  | Rituals like ‘ulma baha’ celebrate the end of the postpartum resting phase. | "We have a ceremony called 'ulma baha,' marking the end of the postpartum resting period with food, cleanliness, and celebration" (FGD 4, Grandmothers)​ |
| Exclusive Breastfeeding | Breastmilk insufficiency and lack of adequate nutrition | "By three months, we give water mixed with sugar and some milk" (FGD 2, Mothers)​ |
| Exclusive Breastfeeding | Breastmilk insufficiency and lack of adequate nutrition | "If my baby cries too much, I will give him milk or porridge to make him full" (FGD 1, Mothers). |
| Exclusive Breastfeeding | Breastmilk insufficiency and lack of adequate nutrition | "If exclusive breastfeeding occurs without a balanced diet, the mother may become physically weak and malnourished... Sometimes, we resort to purchasing milk to supplement breast milk" (FGD 2, Mothers). |
| Exclusive Breastfeeding | Breastmilk insufficiency and lack of adequate nutrition | "We use formula when breastmilk is not enough, but it is expensive and not always available" (FGD 3, Mothers). |
| Exclusive Breastfeeding | Breastmilk insufficiency and lack of adequate nutrition | "If we cannot afford enough nutritious food for the mother, we start giving the baby other food early" (FGD 5, Fathers)​ |
| Exclusive Breastfeeding | Breastmilk insufficiency and lack of adequate nutrition | "Mothers in our community start feeding fenugreek mixed with milk before six months if the baby is hungry" (Traditional Healer)​ |
| Exclusive Breastfeeding | Traditional beliefs | "We advise mothers to breastfeed exclusively, but many follow their family’s traditions instead" (Indepth interview, Healthcare Workers). |
| Exclusive Breastfeeding | Traditional beliefs | “Some mothers think exclusive breastfeeding will make them too weak to care for their other children, so they stop early" (In-depth interview, Healthcare Workers). |
| Other Culturally Important practices | Culturally significant postpartum foods like 'shuro dhalinsaa' (porridge) are prioritized. | "After giving birth, we prepare a dish called 'shuro dhalinsaa' (porridge) for the mother" (FGD 1, Mothers)​ |
| Other Culturally Important practices | Butter is highly valued for its perceived energy-giving properties. | "Butter holds significant importance for mothers, especially after giving birth" (FGD 2, Mothers)​ |
| Economic constraints and lack of nutrition | Wealthier families can provide better maternal nutrition, ensuring sufficient milk production. | "Those who are richer don’t worry about breastfeeding their infants" (FGD 3, Mothers)​ |
| Economic constraints and lack of nutrition | Malnutrition among mothers leads to insufficient breastmilk and reliance on powdered milk. | "We see richer families feeding their babies well, while poorer families struggle" (FGD 5, Fathers)​ |
| Economic constraints and lack of nutrition | Malnutrition among mothers leads to insufficient breastmilk and reliance on powdered milk. | "Some mothers don't produce enough milk because of malnutrition, so they turn to powdered milk or formula" (Traditional Healer, ID_08)​ |
| Economic constraints and lack of nutrition | Malnutrition among mothers leads to insufficient breastmilk and reliance on powdered milk. | "Due to the expense of cow's milk, water, oil, and biscuits are common alternatives" (FGD 1, Mothers)​ |
| Impact of Seasonal Food Insecurity | Seasonal food shortages reduce maternal diet quality, affecting breastfeeding. | "During the dry season, we face many challenges, including feeding our cows to produce milk for the baby" (FGD 2, Mother)​ |
| Socio Cultural influence | Elders strongly influence feeding decisions, often prioritizing cultural wisdom. | "My parents-in-law encourage early initiation of complementary feeding" (In-depth Interview, Mother)​ |
| Socio Cultural influence | Elders strongly influence feeding decisions, often prioritizing cultural wisdom. | "Feeding decisions are often left to grandmothers and mothers-in-law in our culture" (FGD 5, Fathers)​ |
| Knowledge Gaps | Limited education from healthcare providers perpetuates reliance on traditional knowledge. | "I didn't receive any formal education on breastfeeding" (In-depth Interview, Mother)​ |
| Knowledge Gaps | Behavior change efforts by health workers often fail to overcome ingrained practices. | "Despite the advice, many women are not inclined to change behaviors for better health outcomes for themselves and their babies" (FGD 4, Grandmothers)​ |
| Knowledge Gaps | Behavior change efforts by health workers often fail to overcome ingrained practices. | "Health workers focus more on mothers, but fathers should also learn about breastfeeding and child care" (FGD 5, Fathers)​ |
