# Supplementary material for: A mixed-methods investigation of infant and young child feeding practices in rural Ethiopia: integrating insights from surveys, direct observation, and qualitative research
Source: Front Nutr. 2026 Apr 21;13:1794352. doi: 10.3389/fnut.2026.1794352 (PMC13138919; doi:10.3389/fnut.2026.1794352)
Supplement: Supplementary file 3 [file Table_3.docx]

Supplementary Material S1

Semi-Structured Interview and Focus Group Discussion Guides

*A Mixed-Methods Investigation of Infant and Young Child Feeding Practices in Rural Ethiopia: Integrating Insights from Surveys, Direct Observation, and Qualitative Research*

# A. In-depth interview guides

## A1. Mothers

1. What customs or traditions did you follow when feeding your baby in the first days after birth?
2. Did you give your baby any foods or liquids before breastfeeding was established? If yes, what was given, who suggested it, and why?
3. How was your breastfeeding experience in the early days after birth? What helped, and what made breastfeeding difficult?
4. How did you decide when to start giving your baby foods or liquids other than breast milk?
5. How did your husband, mother-in-law, grandmother, or other family members influence feeding decisions?
6. Did you receive any advice or education about infant feeding? If yes, from whom, and what advice did you receive?
7. How did food availability, household work, or seasonal conditions affect breastfeeding or other feeding practices?
8. Can you describe the support you received during the postpartum resting period (ulma) and how it affected feeding your baby?

## A2. Community influencers and leaders

This guide was used with grandmothers and community influencers, including traditional birth attendants, religious leaders, traditional healers, and health workers.

1. From your perspective, what cultural beliefs or traditions influence how newborns and young infants are fed in this community?
2. What are the common reasons families give foods or liquids before breastfeeding is established?
3. What challenges do mothers face in breastfeeding exclusively during the first 6 months?
4. What foods or liquids are commonly given to infants before 6 months, and for what reasons?
5. What advice or messages do families receive about breastfeeding and complementary feeding?
6. How do religious, traditional, or health-related messages influence infant feeding decisions?
7. How do seasonal constraints, maternal workload, or household food insecurity affect infant feeding practices?

# B. Focus group discussion guides

## B1. Mothers and elder caregivers

1. What cultural practices and foods are considered important after childbirth for maternal recovery and breastfeeding?
2. What are the common beliefs and practices around colostrum and prelacteal feeding?
3. How do families decide when to begin foods or liquids other than breast milk?
4. How do food availability, season, and socioeconomic conditions affect infant feeding practices?
5. How can families and communities better support mothers to practice optimal infant feeding?
6. What health messages about infant feeding are most trusted, and what messages need more attention?

## B2. Fathers

1. What practices do fathers encourage during pregnancy and after childbirth to support mothers and infants?
2. What challenges do mothers face in exclusively breastfeeding, and how can fathers help?
3. How are decisions made about giving foods or liquids other than breast milk?
4. How do work demands, food shortages, or seasonal pressures affect infant feeding in households?
5. Which community members are most influential in changing infant feeding practices, including prelacteal feeding and breastfeeding?

**Abbreviation:** FGD, focus group discussion.
